# Extracellular vesicles derived from M1 macrophages deliver miR-146a-5p and miR-146b-5p to suppress trophoblast migration and invasion by targeting TRAF6 in recurrent spontaneous abortion

**DOI:** 10.7150/thno.58731

**Published:** 2021-03-31

**Authors:** Jinli Ding, Yan Zhang, Xiaopeng Cai, Yi Zhang, Sisi Yan, Jiayu Wang, Sainan Zhang, Tailang Yin, Chaogang Yang, Jing Yang

**Affiliations:** 1 Reproductive Medical Center, Renmin Hospital of Wuhan University & Hubei Clinic Research Center for Assisted Reproductive Technology and Embryonic Development, Wuhan 430060, China.; 2 Department of Clinical Laboratory, Renmin Hospital of Wuhan University, Wuhan 430060, China.; 3 Department of Gastrointestinal Surgery, Zhongnan Hospital of Wuhan University, Wuhan 430071, China.

**Keywords:** recurrent spontaneous abortion, M1 macrophages, trophoblasts, extracellular vesicles, TRAF6

## Abstract

**Rationale:** Emerging evidence demonstrates that insufficient migration and invasion of trophoblasts play critical roles in the pathogenesis of recurrent spontaneous abortion (RSA). Cell-to-cell communication at the maternal-fetal interface is essential to maintain the invasion and migration of trophoblasts. M1 macrophages, important immune cellular components at the maternal-fetal interface, have been reported to be elevated in decidua tissues from patients with RSA. Recent studies indicate that M1 macrophages modulate trophoblast biological behaviors; however, the underlying mechanisms remain poorly understood.

**Methods:** Extracellular vesicles (EVs) were isolated from the supernatant of M1 macrophages inducted from THP-1 cells (M1-EVs) by ultracentrifugation, identified by transmission electron microscopy, nanoparticle tracking analysis, and western blotting, and their miRNA profile was characterized by miRNA sequencing. Scratch wound healing and transwell assays were used to investigate the effect of M1-EVs on trophoblast migration and invasion. RT-PCR, western blotting, and luciferase reporter assays were conducted to uncover the underlying mechanism. Finally, animal experiments were employed to explore the effect of M1-EVs on embryo absorption in mice.

**Results:** M1 macrophages suppressed trophoblast EMT to reduce their migration and invasion abilities *in vitro* by secreting EVs. Through miRNA sequencing, miR-146a-5p and miR-146b-5p were identified as the most upregulated miRNAs in trophoblasts treated with M1-EVs. Further functional experiments showed that M1-EVs inhibited trophoblast migration and invasion by transferring miR-146a-5p and miR-146b-5p. Mechanistically, EV miR-146a-5p and miR-146b-5p inhibited EMT of trophoblasts by directly suppressing TNF receptor-associated factor 6 (TRAF6) expression at the post-transcriptional level. Furthermore, M1-EVs aggravated embryo absorption in mice. Clinically, expression of miR-146a-5p, miR-146b-5p, and TRAF6 were aberrant in placental villous tissues from patients with RSA, and negative correlations were found between miR-146a-5p/miR-146b-5p and TRAF6 expression levels.

**Conclusions:** Our findings indicate that miR-146a-5p and miR-146b-5p derived from EVs play important roles in intercellular communication between M1 macrophages and trophoblasts, illuminating a novel mechanism in M1 macrophage regulation of trophoblasts and their role in RSA.

## Introduction

Recurrent spontaneous abortion (RSA), defined as two or more consecutive pregnancy losses before 20 weeks of gestation, occurs in 2-5% of women during their reproductive years 1. As a complex pathological process, RSA involves multiple factors and diverse cell components at the maternal-fetal interface 2-4. Extravillous trophoblasts (EVTs), which are derived from invaded trophoblasts, are an important part of the placenta and exert vital roles in embryo implantation and pregnancy 5. EVTs migrate away from the attached embryo and invade the uterine epithelium and spiral arteries, thereby establishing the maternal-fetal linkage and providing signals to ensure maternal immune cells remain resistant to the embryo 6-8. Ours and other previous studies have indicated that insufficient EVT migration and invasion usually cause the failed connection between mother and fetus that is involved in RSA 2, 5, 9. Epithelial-mesenchymal transition (EMT), characterized by loss of epithelial phenotype and acquisition of mesenchymal phenotype, plays an important role in the regulation of EVT migration and invasion 10. A number of researchers have proposed that EMT is part of the EVT differentiation process 11, 12. Therefore, exploration of the potential mechanisms underlying EMT of EVTs has great significance for further understanding the pathogenesis of RSA.

Emerging and accumulating evidence indicates that the initiation and maintenance of trophoblast EMT are closely related to the immune microenvironment at the maternal-fetal interface 10, 13. Macrophages, as the second largest type of leukocytes at the maternal-fetal interface, play an important role in regulating embryo implantation and pregnancy 14, 15. Depending on environmental cues, macrophages can be polarized into two subpopulations: classic M1 (M1-Mφ) and alternative M2 (M2-Mφ) macrophages. M1-Mφ mainly have pro-inflammatory effects, while M2-Mφ act as anti-inflammatory macrophages 16. Increasing evidence indicates that the polarization balance between M1-Mφ and M2-Mφ plays an important role in trophoblast invasion and migration. Our previous studies have also confirmed that M2-Mφ can establish dialogue with trophoblasts to participate in normal pregnancy. Trophoblasts can promote macrophage polarization to M2 phenotype by secreting IL-6 17. Additionally, M2-Mφ can promote EMT of trophoblasts by secreting G-CSF, thereby enhancing their invasion and migration capabilities 18. Numerous studies have shown that macrophages in RSA exhibit abnormal polarization status, which manifests as a high proportion of M1-Mφ or a decline in M2-Mφ in the decidua 3, 4, 19, 20. It was also reported that M1-Mφ can inhibit the invasion and migration of trophoblasts by secreting cytokines such as TNF-α 14. Although crosstalk between M1-Mφ and trophoblasts has been established, the potential mechanisms underlying M1-Mφ regulation of trophoblast EMT are still unclear.

In addition to soluble cytokines, extracellular vesicles (EVs) are important mediators of cell-to-cell communication 21, 22. EVs are membrane-bound vesicles 40-100 nm in diameter that are released by most cell types including macrophages 22, 23. Growing evidence demonstrates that EVs are capable of transferring their contents, including proteins and various nucleic acids (mRNAs, miRNAs, lncRNAs, circRNAs), to recipient cells to regulate their biological behaviors 24-26. Recently, EVs were also reported to play important roles in maintenance of placental homeostasis 27. Given the interactions between macrophages and trophoblasts at the maternal-fetal interface and the important roles of EVs in mediating intercellular communication, we speculated that M1-Mφ may inhibit EMT of trophoblasts by secreting EVs that reduce their invasion and migration capabilities, thereby participating in the occurrence of RSA.

To investigate the above hypothesis, we conducted systematic experiments *in vitro*, *in vivo*, and using clinical samples. Our *in vitro* results show that EVs from M1-Mφ (M1-EVs) were transferred into trophoblasts and inhibited their invasion and migration abilities via regulating EMT. We identified miRNA-146a-5p and miRNA-146b-5p as the critical miRNAs inhibiting trophoblast EMT by comparing miRNAs in M1-EVs-treated trophoblasts and normal trophoblasts. Mechanistically, miR-146a-5p and miR-146b-5p from M1-EVs inhibited EMT by directly suppressing TNF receptor-associated factor 6 (TRAF6) to decrease trophoblast invasion and migration. Furthermore, M1-EVs were demonstrated to increase embryo absorption in mice. Clinically, expression of miR-146a-5p, miR-146b-5p, and TRAF6 were aberrant in placental tissues from patients with RSA, and negative correlations were found between miR-146a-5p/miR-146b-5p and TRAF6. Collectively, our findings indicate that EV miR-146a-5p and miR-146b-5p play important roles in the communication between M1-Mφ and trophoblasts, illuminating a novel mechanism underlying M1-Mφ regulation of EVTs and their role in RSA.

## Materials and Methods

### Patients and tissue samples

Placental villous tissues were obtained from 20 healthy women (induced abortion for unwanted pregnancy) and 34 patients with RSA at Renmin Hospital of Wuhan University (Wuhan, China) between December 2017 and October 2019. Women presenting the following criteria were excluded from the study: (a) symptoms of endocrine or metabolic diseases, (b) abnormal karyotype analysis, or (c) uterine abnormality. RSA was defined as the sequential loss of two or more pregnancies before 20 weeks of pregnancy. Patients with RSA with chromosomal abnormalities in the embryo were excluded from the study. Some of the tissues were fixed in 4% paraformaldehyde for paraffin embedding in blocks, and the rest were frozen and stored in liquid nitrogen. All samples were collected with informed consent from the patients, and all related procedures were performed with the approval of the internal review and ethics boards of Renmin Hospital of Wuhan University. The baseline characteristics of the patients are summarized in **Table S1**.

### Cell culture and reagents

The trophoblast cell lines HTR-8/SVneo (HTR-8) and JEG3 were grown in DMEM/F-12 medium (Gibco, USA), the human monocyte cell line THP-1 was cultured in PRIM-1640 medium (Gibco), and Raw 264.7 macrophages were grown in DMEM/high glucose medium (Gibco) supplemented with 10% fetal bovine serum (FBS) (Gibco) at 37 °C in 5% CO_2_. For M0 macrophage polarization, 5 × 10^5^/mL THP-1 cells were cultured in PRIM-1640 with 50 ng/mL phorbol 12-myristate 13-acetate (PMA; Sigma-Aldrich, USA) for 24 h. For M1 macrophages, PMA-stimulated THP-1 cells or Raw 264.7 macrophages were stimulated with 100 ng/mL lipopolysaccharide (LPS; Sigma-Aldrich) and 20 ng/mL IFN-γ (PeproTech, USA), while IL-4 and IL-13 were applied to stimulate for M2 macrophages 16, 28.

### EV preparation and treatment

EVs were collected from the supernatant of M1-Mφ (cultured in EV-free medium) and isolated by ultracentrifugation. Briefly, cell culture supernatant was processed (300 ×*g*, 10 min; 2000 ×*g*, 10 min; 10,000 ×*g*, 30 min), centrifuged (100,000 ×*g*, 70 min), washed with phosphate-buffered saline (PBS), and further centrifuged (100,000 ×*g*, 70 min) 29. The retained EVs were then used to stimulate cells. Cells were plated onto 6-well plates, grown to ~50% confluence, and then treated with 100 μg/mL EVs or PBS as control. The cells were collected for subsequent experiments 48 h later.

### Blockade of EV generation

GW4869 (Sigma-Aldrich) was used to inhibit EV release. 10 mM GW4869 was added to the medium of M1 macrophages with 10% EV-free FBS. The conditioned medium was collected 48 h later for EV isolation, as described above.

### Transmission electron microscopy (TEM)

TEM was used to identify the morphology of EVs. EVs were prepared according to the manufacturer's instructions. EVs were fixed in 1% glutaraldehyde and then washed with deionized water. EV suspension was placed on formvar carbon-coated 300-mesh copper electron microscopy grids (Agar Scientific, UK) and incubated at room temperature for 5 min. Then, the EVs were negatively stained with 2% uranyl oxalate at room temperature for 1 min. The grids were washed with PBS and air dried for 5 min. Images were obtained by TEM (JEM-100CX-II, JEOL, Japan).

### Nanoparticle tracking analysis (NTA)

The size distribution and concentration of M1-EVs were analyzed by NTA according to the manufacturer's instructions (NanoSight Technology, Malvern, UK), as previously reported 30.

### Cellular internalization of EVs

M1-EVs were labelled with PKH67 (Sigma-Aldrich). Briefly, M1-EVs were resuspended in Diluent C mixed with PKH67, incubated at room temperature, and the reaction was stopped with EV-free FBS. Excess dye was removed by centrifuging the EVs at 100,000 ×*g* for 60 min. The labelled EVs were resuspended in PBS and incubated with 60% confluent HTR-8 or JEG3 cells for 12, 24, and 48 h. The cells were washed with PBS three times and fixed with 4% paraformaldehyde at room temperature for 30 min. The cells were then stained with DilC_16_. A fluorescence microscope (BX53, Olympus, Japan) was used to observe internalized PKH67-labelled M1-EVs.

### miRNA sequencing of EVs

Total RNA was isolated from M1-EVs using Total EVs RNA Isolation Kit (RiboBio, China). HTR-8 cells treated with M1-EVs for 48 h were compared with HTR-8 cells cultured in DMEM/F-12 medium (control). RiboBio tested the quality and amount of miRNA and constructed and sequenced the miRNA library. A HiSeq 2500 System (Illumina, USA) was used to sequence the library, and Illumina analysis software was applied to the raw reads.

### Cell transfection

The adenovirus for TRAF6 (Ad-TRAF6) and a negative control (Ad-control) were purchased from Hanheng Biotechnology (China). Adenovirus transfection was conducted when cells reached 50-60% confluence. miRNA mimics and inhibitors of miRNA-146a-5p and miRNA-146b-5p, siRNA for TRAF6, and the corresponding primers were synthesized by RiboBio. HTR-8 and JEG3 cells were seeded on 6-well plates 24 h before transfection. When the cells reached 40-50% confluence, miRNA mimics, inhibitors, or siRNA were transfected with Lipofectamine 2000 (Invitrogen, USA) according to the manufacturer's instructions. 48 h after transfection, the cells were harvested for subsequent experiments.

### RNA isolation and quantitative real-time PCR (RT-PCR)

Total RNA was isolated using TRIzol Reagent (Invitrogen) according to the manufacturer's instructions. For mRNA expression, cDNA was synthesized with mRNA Reverse-Transcription Kit (Takara, Japan). For miRNA expression, reverse-transcription experiments were performed with Bulge-Loop^TM^ miRNA RT-PCR Starter Kit (RiboBio). GAPDH or U6 were used for normalization. SYBR Green PCR Mix (Takara) was used for quantitative assays with a 7500 Real-Time PCR System (Applied Biosystems, USA). The 2^-ΔΔCt^ method was used to calculate the relative mRNA and miRNA expression levels. miRNA primers were provided by RiboBio. The primer sequences are presented in **Table S2**.

### Western blotting

Protein extraction and western blotting were performed as previously reported 2. The following primary antibodies were obtained from Proteintech (USA): anti-vimentin (Cat# 10366-1-AP), anti-E-cadherin (Cat# 20874-1-AP), anti-N-cadherin (Cat# 22018-1-AP), anti-TRAF6 (Cat# 66498-1-Ig), and anti-GAPDH (Cat# 10494-1-AP).

### Scratch wound healing assay

To assess cell motility, a scratch wound healing assay was conducted. HTR-8 or JEG3 cells were seeded on 6-well plates and cultured to 100% confluence. Scratches were made with 200 μL pipette tips and then the wounded monolayers were washed with PBS and incubated in serum-free medium. Wound healing rates were determined at 48 h using an inverted microscope (BX53, Olympus). The images were analyzed and quantified using ImageJ (NIH, USA).

### Transwell assay

A transwell system with 0.4 μm chambers (Corning, USA) was used to study the invasive ability of trophoblasts. The assay was performed according to a previously described method 2. Invaded cells were imaged under a microscope (Olympus) and quantitated by counting cells in 5 random fields on the lower membrane surface.

### Dual-luciferase reporter assay

The 3'-UTR sequence of TRAF6 gene (wild-3'-UTR) and miR-146a-5p/miR-146b-5p binding sites were amplified and then sub-cloned into the p-MIR-reporter plasmid (Ambion, USA). The wild-type 3'-UTR of TRAF6 containing the mutant miR-146a-5p/miR-146b-5p binding site sequences was mutated (from CAGUAUUA to CUGAAAUA, mut-3'-UTR) and inserted into an equivalent luciferase reporter plasmid. HTR-8 cells were cotransfected with the mutant or wild-type 3'-UTR reporter and miR-146a-5p/miR-146b-5p mimics/inhibitors. Renilla luciferase reporter vector Prl-SV40 (Promega, USA) was provided as an internal transfection control. 48 h after transfection, the total cell lysates were harvested and the luciferase activity of Renilla was detected by Dual-Luciferase Reporter Assay System (Promega) according to the manufacturer's instructions.

### *In situ* hybridization (ISH) and immunohistochemistry (IHC)

ISH was performed to detect miR-146a-5p and miR-146b-5p in the placental villous tissues of patients with RSA and healthy women using ISH kits (Exon Biological Technology, China) on 4 μm paraffin sections. ISH was performed following previously described methods 31. Serial sections from the placental villous tissues were obtained for IHC, which was conducted following previously described methods 32.

### Animal experiments

Eight-week-old female C57BL/6 and male BALB/c mice were obtained from the Animal Experiment Center of Wuhan University. The female mice were divided into two groups: normal (n = 5) and M1-EVs (n = 5). M1-EVs were obtained from the supernatants of Raw 264.7 macrophages stimulated with LPS and IFN-γ. The female mice were mated with the male mice at a ratio of 2:1. After setting a concentration gradient for pre-experiments, M1-EVs with a volume of 100 mg or PBS were injected into the female mice via the tail vein on the day of plug detection (day 0.5). The injection was repeated once every three days, and the mice were executed on day 11.5. Embryo resorption was calculated, and the expression levels of miR-146a-5p, miR-146b-5p, and TRAF6 were analyzed.

### Statistics analysis

All *in vitro* experiments were performed independently at least 3 times. All statistical analyses were performed with SPSS statistical software (version 22.0, IBM SPSS, USA). Groups of discrete variables were compared by Kruskal-Wallis nonparametric analysis of variance or Mann-Whitney *U* test. *P* values < 0.05 were considered statistically significant.

## Results

### M1-Mφ suppress EMT, migration, and invasion of trophoblasts *in vitro*

To explore the effects of M1-Mφ on the migration and invasion of trophoblasts, M1-Mφ were obtained by treating PMA-induced THP-1 cells with LPS and IFN-γ (Figure S1A). The cell morphology changed from rounded to multiple non-rounded shapes after treatment (Figure S1B). To further confirm polarization to M1-Mφ, we examined the expression of M1 markers and found that the mRNA expression levels of IL-6, TNF-α and IFN-β were significantly upregulated after LPS and IFN-γ treatment (Figure S1C), with similar results at the protein level of iNOS (Figure S1D). In addition, flow cytometry analysis indicated that the stimulation increased the expression levels of the M1 surface markers CD80 and CD86 (Figure S1E). Collectively, these results identified the successful induction of M1-Mφ.

**Figure 1:**
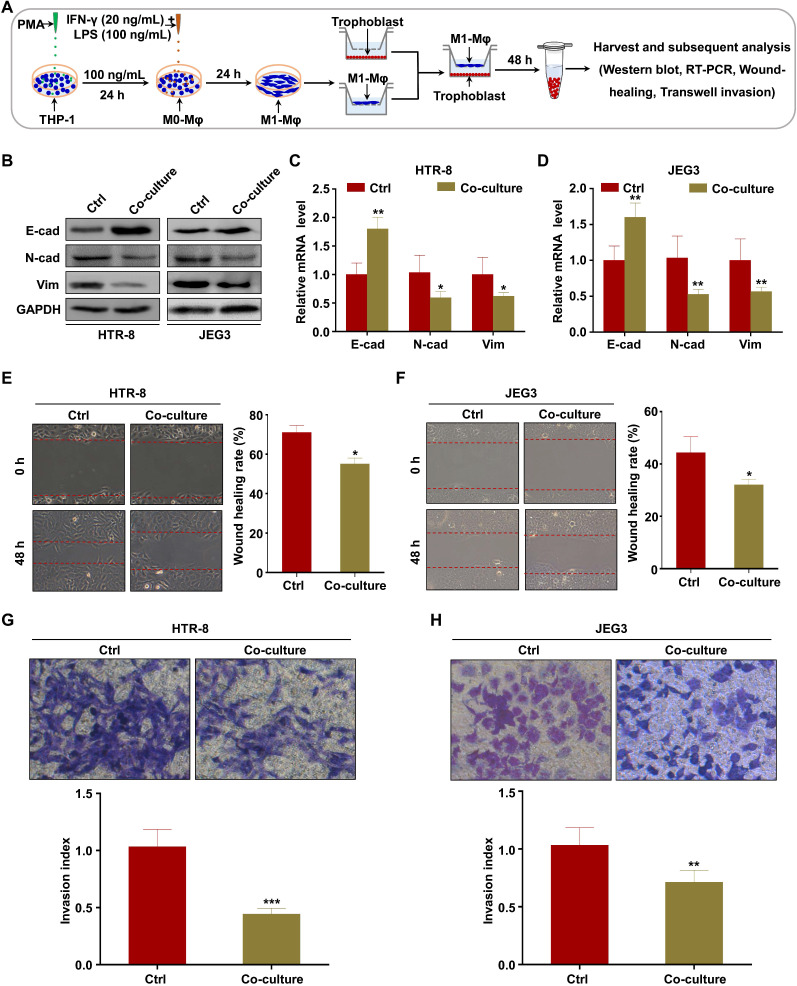
** M1-Mφ suppress EMT, migration, and invasion of trophoblasts *in vitro*.** (**A**) Schematic illustration of the M1-Mφ and trophoblast co-culture model and the experimental design. (**B**) Western blot analysis of E-cadherin (E-cad), N-cadherin (N-cad), and vimentin (Vim) protein levels in HTR-8 and JEG3 cells co-cultured with M1-Mφ or control. (**C-D**) RT-PCR assays of E-cad, N-cad, and Vim mRNAs in HTR-8 and JEG3 cells co-cultured with M1-Mφ or control. (**E-H**) Migration and invasion capacities of trophoblasts (HTR-8 and JEG3) alone or co-cultured with M1-Mφ determined by wound healing and transwell assays, respectively. Representative images of migrated or invaded cells are shown (magnification, × 200). Error bars, SD. *P < 0.05, **P < 0.01.

To investigate whether M1-Mφ regulate EMT of trophoblasts *in vitro*, HTR-8 and JEG3 cells were co-cultured with M1-Mφ in a non-contact transwell system, in which soluble factors are exchanged but cells are impermeable (Figure 1A). Western blotting and RT-PCR were performed to analyze EMT markers. We found that expression of the epithelial marker E-cadherin was increased, while the mesenchymal markers N-cadherin and vimentin were downregulated in both HTR-8 and JEG3 cells after co-culture with M1-Mφ (Figure 1B-D). Wound healing and transwell assays were conducted to determine if M1-Mφ affected the migration and invasion abilities of the trophoblasts. Trophoblasts co-cultured with M1-Mφ displayed slower wound closure (Figure 1E-F), and were less invasive (Figure 1G-H) compared with control. Taken together, our findings suggest that M1-Mφ suppress EMT and inhibit the migration and invasion abilities of trophoblasts *in vitro*.

### M1-Mφ suppress EMT, migration, and invasion of trophoblasts via secreting EVs

**Figure 2:**
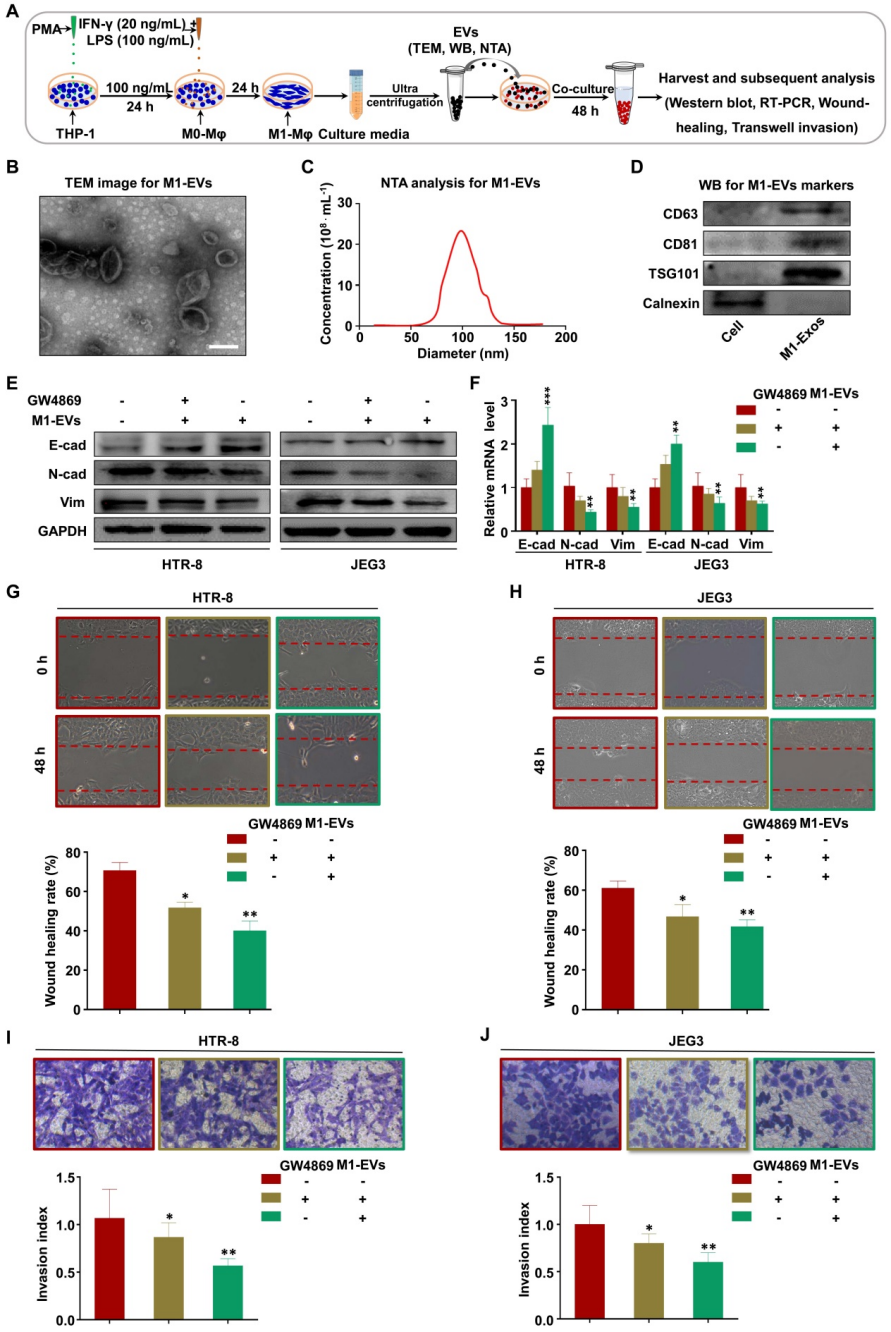
** M1-Mφ suppress EMT, migration, and invasion of trophoblasts via secreting EVs.** (**A**) Schematic illustration of the EV acquisition method and the experimental design. (**B**) Representative TEM image of M1-EVs with a lipid bilayer structure (Scale bar, 100 nm). (**C**) NTA of the size distribution and concentration of M1-EVs. (**D**) Western blot analysis of EV fractions and cell lysates of M1-Mφ with antibodies against exosomal proteins (CD63, CD81, TSG101) and the cellular protein calnexin. (**E-F**) Western blot and RT-PCR analysis of E-cadherin (E-cad), N-cadherin (N-cad), and vimentin (Vim) protein and mRNA levels in HTR-8 and JEG3 cells treated with M1-EVs or an equal volume of medium from M1-Mφ treated with GW4869. (**G-J**) Cell migration and invasion capacities of trophoblasts (HTR-8 and JEG3) treated with control, M1-EVs, or EVs from an equal volume of medium from M1-Mφ treated with GW4869 determined by wound healing and transwell assays, respectively. Representative images of migrated or invaded cells are shown (magnification, × 200). Error bars, SD. *P < 0.05, **P < 0.01, ***P < 0.001.

Recent studies have demonstrated that macrophages secrete EVs in large amounts and transfer signaling molecules to surrounding cells 33, 34. We hypothesized that EVs from M1-Mφ (M1-EVs) might negatively mediate the migration and invasion of trophoblasts in our experimental system (Figure 2A). To verify this, we isolated EVs from the conditioned media of M1-Mφ by ultracentrifugation. The M1-EVs were characterized to be round particles with a bilayer membrane and 50-150 nm diameter by TEM (Figure 2B) and NTA (Figure 2C). Western blotting showed that the specific markers CD63, CD81, and TSG101 were abundant in the M1-EVs but not detected in whole cell lysate, while calnexin was detected in the cell lysate but not in the M1-EVs (Figure 2D). Based on these results, we confirmed that the isolated substances were EVs.

Next, we investigated whether M1-EVs mediate EMT of trophoblasts *in vitro*. Treatment of trophoblasts with M1-EVs induced expression of E-cadherin and inhibited expression of N-cadherin and vimentin (Figure 2E-F). To further explore whether EVs have a critical role in this effect, we blocked EV formation by treating M1-Mφ with GW4869 (the secretory-specific inhibitor of EVs 35-37), and EVs from equal volume of culture medium were collected to treat HTR-8 and JEG3 cells. NTA analysis confirmed that GW4869 decreased secretion of EVs in the supernatant (Figure S2). Additionally, M1-Mφ failed to inhibit EMT of trophoblasts following GW4869 treatment (Figure 2E-F). Further functional experiments demonstrated that trophoblasts treated with M1-EVs displayed slower wound closure and less invasion compared with control, which were reversed after GW4869 treatment (Figure 2G-J). Collectively, these results demonstrate that M1-Mφ suppress EMT, migration, and invasion of trophoblasts via secreting EVs.

### M1-EVs transport miR-146a-5p and miR-146b-5p into trophoblasts

**Figure 3:**
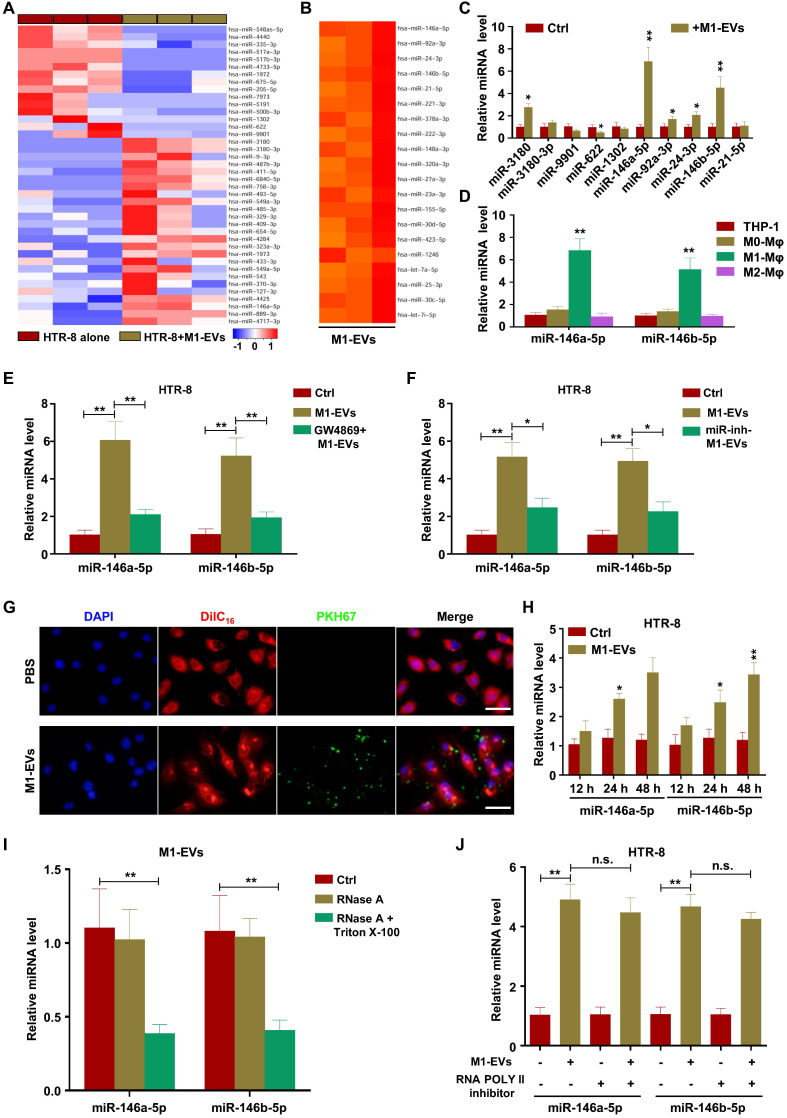
** M1-EVs transport miR-146a-5p and miR-146b-5p into trophoblasts.** (**A**) Heat maps showing the relative expression levels of miRNAs in HTR-8 cells and HTR-8 cells treated with M1-EVs. (**B**) Heat maps showing the relative expression levels of miRNAs in M1-EVs. (**C**) RT-PCR assays of miRNAs in HTR-8 cells treated with M1-EVs or control. miRNA levels are normalized to the control group. (**D**) Expression levels of miR-146a-5p and miR-146b-5p measured by RT-PCR in EVs of THP-1, M0, M1, and M2 macrophages. (**E**) Expression levels of miR-146a-5p and miR-146b-5p measured by RT-PCR in HTR-8 cells treated with control, M1-EVs, or EVs from an equal volume of medium from M1-Mφ treated with GW4869. (**F**) Expression levels of miR-146a-5p and miR-146b-5p measured by RT-PCR in HTR-8 cells treated with control, M1-EVs, or EVs from M1-Mφ treated with inhibitors of miR-146a-5p or miR-146b-5p. (**G-H**) Confocal fluorescence microscopy images of HTR-8 cells incubated with PKH67-labeled M1-EVs (green) for 12, 24, and 48 h (scale bar, 20 µm). The expression levels of miR-146a-5p and miR-146b-5p were measured by RT-PCR. (**I**) RT-PCR analysis of miR-146a-5p and miR-146b-5p expression in M1-EVs treated with RNase A alone or in combination with Triton X-100. (**J**) Expression levels of miR-146a-5p and miR-146b-5p measured by RT-PCR in HTR-8 cells treated with polymerase II inhibitors and then incubated with M1-EVs. Error bars, SD. *P < 0.05, **P < 0.01; n.s., not significant.

miRNAs have demonstrated important roles in EV-mediated pathological regulation 38. Therefore, to determine the possible mechanisms underlying M1-EV suppression of trophoblast migration and invasion, we sequenced miRNAs from M1-EVs, HTR-8 cells treated with M1-EVs, and HTR-8 cells alone. The analysis revealed that numerous miRNAs had altered expression in HTR-8 cells following M1-EVs treatment, with miR-3180, miR-3180-3p, miR-9901, miR-622, and miR-1302 having the largest differences (Figure 3A). In addition, miR-146a-5p, miR-92a-3p, miR-24-3p, miR-146b-5p, and miR-21-5p were identified as the most abundant miRNAs in M1-EVs (Figure 3B and Table S3). The above ten miRNAs were further verified in dependent samples by RT-PCR. The results demonstrated that miR-146a-5p and miR-146b-5p were the most upregulated miRNAs in M1-EVs-treated HTR-8 (Figure 3C) and JEG3 (Figure S3A) cells. Therefore, M1-EVs might regulate the biological behaviors of trophoblasts by transferring miR-146a-5p and miR-146b-5p. To determine whether M1-Mφ were the main source of miR-146a-5p and miR-146b-5p, the expression levels of miR-146a-5p and miR-146b-5p in EVs of macrophages in different polarization states were measured by RT-PCR. The results indicated that M1-Mφ displayed the highest levels of these miRNAs (Figure 3D). To further clarify whether EVs mediated the inhibitory effect of M1-Mφ, HTR-8 and JEG3 cells were treated with EVs collected from M1-Mφ treated with GW4869. The results showed that GW4869 markedly reduced the ability of M1-EVs to increase the expression levels of miR-146a-5p and miR-146b-5p in HTR-8 (Figure 3E) and JEG3 (Figure S3B) cells. In addition, M1-Mφ were transfected with inhibitors of miR-146a-5p or miR-146b-5p and EVs were extracted to treat HTR-8 and JEG3 cells. The results showed that EVs from M1-Mφ transfected with miR-146a-5p or miR-146b-5p inhibitors decreased the expression levels of miR-146a-5p or miR-146b-5p, respectively, in HTR-8 (Figure 3F) and JEG3 (Figure S3C) cells compared with EVs from M1-Mφ.

To determine if M1-EVs were internalized by HTR-8 and JEG3 cells, M1-EVs were purified and labeled with PKH67 and incubated with HTR-8 and JEG3 cells for 12, 24, and 48 h. We observed that PKH67-labeled M1-EVs were internalized by HTR-8 (Figure 3G) and JEG3 (Figure S3D) cells at 12 h. Compared with the control group, M1-EVs significantly promoted expression of miR-146a-5p and miR-146b-5p in HTR-8 (Figure 3H) and JEG3 (Figure S3E) cells at 24 and 48 h, suggesting that miR-146a-5p and miR-146b-5p were transferred from M1-Mφ to trophoblasts via EVs. In addition, the levels of miR-146a-5p and miR-146b-5p in M1-EVs were significantly decreased following treatment with RNase A and Triton X-100 but unchanged after RNase A treatment alone (Figure 3I), indicating that extracellular miR-146a-5p and miR-146b-5p were mainly encased within the membrane instead of directly released. In addition, pretreatment with RNA polymerase II inhibitor did not affect the expression levels of miR-146a-5p and miR-146b-5p in recipient HTR-8 (Figure 3J) and JEG3 (Figure S3F) cells treated with M1-EVs, indicating that enhancement of cellular miR-146a-5p and miR-146b-5p in trophoblasts arose from M1-EVs-mediated miRNA transfer, not endogenous miR-146a-5p and miR-146b-5p induction. In order to validate the effects of miR-146a-5p and miR-146b-5p delivered by EVs on trophoblasts, miR-146a-5p or miR-146b-5p were knocked down in M1-Mφ by corresponding adenoviruses and the EVs were collected for trophoblast treatment. The results indicated that the adenoviruses significantly downregulated miR-146a-5p or miR-146b-5p (Figure S4A). Depletion of miR-146a-5p partially rescued the inhibitory effect of M1-EVs on EMT of HTR-8 and JEG3 cells (Figure S4B-C), and similar results were observed for miR-146b-5p (Figure S4D-E). These findings reveal that functional miR-146a-5p and miR-146b-5p are transferred from M1-Mφ to trophoblasts via EVs.

### MiR-146a-5p and miR-146b-5p suppress EMT, migration, and invasion of trophoblasts

**Figure 4:**
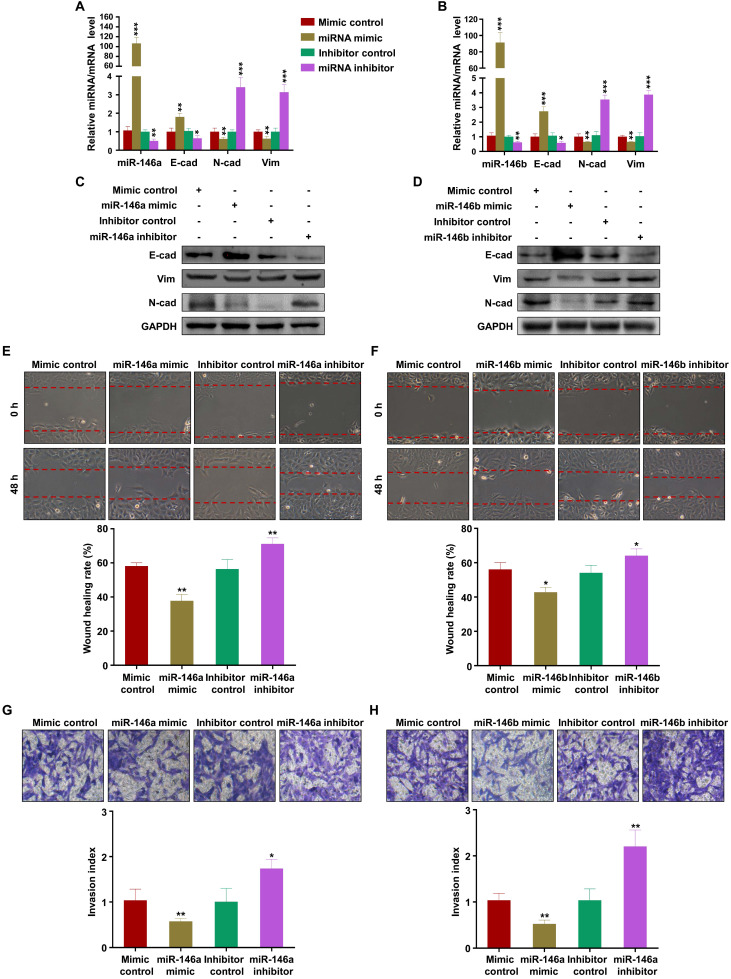
** miR-146a-5p and miR-146a-5p suppress EMT, migration, and invasion of trophoblasts *in vitro*.** (**A-B**) Expression levels of miR-146a-5p or miR-146b-5p and E-cadherin (E-cad), N-cadherin (N-cad), and vimentin (Vim) mRNA measured by RT-PCR in HTR-8 cells 48 h after transfection with miR-146a-5p or miR-146b-5p mimics or inhibitors, respectively. (**C-D**) Expression levels of E-cad, N-cad, and Vim protein measured by western blotting 48 h post transfection. (**E-H**) Migration and invasion capacities of HTR-8 cells treated with control or M1-EVs determined by wound healing and transwell assays, respectively. Representative images of migrated or invaded cells are shown (magnification, × 200). Error bars, SD. *P < 0.05, **P < 0.01, ***P < 0.001.

To verify the effects of miR-146a-5p and miR-146b-5p on the migration and invasion of trophoblasts, we transfected mimics or inhibitors of miR-146a-5p or miR-146b-5p into HTR-8 and JEG3 cells. The results showed that miR-146a-5p and miR-146b-5p mimics promoted expression of E-cadherin and inhibited expression of N-cadherin and vimentin, while the inhibitors had opposite effects, in HTR-8 (Figure 4A-D) and JEG3 (Figure S5A-D) cells. Wound healing and transwell assays demonstrated that miR-146a-5p and miR-146b-5p mimics slowed wound closure in HTR-8 (Figure 4E-F) and JEG3 (Figure S5E-F) cells and reduced invasion of HTR-8 (Figure 4G-H) and JEG3 (Figure S5G-H) cells compared with control, while the inhibitors displayed opposite effects. Collectively, these results indicate that EVs derived from M1-EVs restrict the migration and invasion of trophoblasts by transferring miR-146a-5p or miR-146b-5p into trophoblasts.

### TRAF6 is the common target of miR-146a-5p and miR-146b-5p

**Figure 5:**
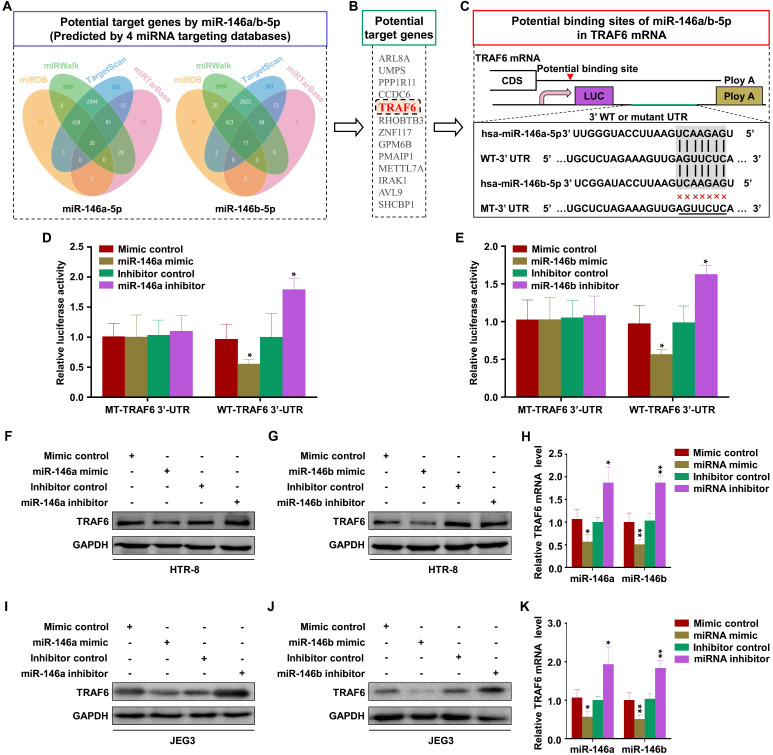
** TRAF6 is the common target of miR-146a-5p and miR-146b-5p.** (**A**) Four independent miRNA target databases were used to predict potential miRNAs. (**B**) Common target genes of miR-146a-5p and miR-146b-5p. (**C**) Schematic representation of the TRAF6 3'-UTR. Mutations were generated at the predicted miR-146a-5p and miR-146b-5p-binding sites. (**D**) Relative luciferase activity in HTR-8 cells cotransfected with MT-TRAF6 3'-UTR + miR-146a-5p control, MT-TRAF6 3'-UTR + miR-146a-5p mimic, WT-TRAF6 3'-UTR + inhibitor control, or WT-TRAF6 3'-UTR + miR-146a-5p inhibitor. (**E**) Relative luciferase activity in HTR-8 cells cotransfected with MT-TRAF6 3'-UTR + miR-146b-5p control, MT-TRAF6 3'-UTR + miR-146b-5p mimic, WT-TRAF6 3'-UTR + inhibitor control, or WT-TRAF6 3'-UTR + miR-146b-5p inhibitor. (**F-H**) Levels of TRAF6 mRNA and protein measured by RT-PCR and western blotting in HTR-8 cells 48 h after transfection with miR-146a-5p or miR-146b-5p mimics or inhibitors. (**I-K**) Levels of TRAF6 mRNA and protein measured by RT-PCR and western blotting in JEG3 cells 48 h after transfection with miR-146a-5p or miR-146b-5p mimics or inhibitors. Error bars, SD. *P < 0.05, **P < 0.01.

Numerous studies have indicated that miRNAs exert their biological function mainly by regulating the expression of downstream target genes 39. Thus, the public databases miRWalk, TargetScan, miRTarBase, and miRDB were used to predict the target genes of miR-146a-5p and miR-146b-5p (Figure 5A). The analysis identified 13 common target genes of miR-146a-5p and miR-146b-5p (Figure 5B). Then, literature review and David Bioinformatics Resources were used to analyze the functional annotation clustering of these genes. TRAF6 has been reported to be related to cell invasion and migration 40, 41. Further, an miRNA online database prediction showed that there are potential binding sites for miR-146a-5p and miR-146b-5p in the TRAF6 3'-UTR (Figure 5C). To confirm whether TRAF6 is a common direct target of miR-146a-5p and miR-146b-5p, wild-type or mutant miRNA binding site TRAF6 3'-UTR-driven luciferase vectors were co-transfected into HTR-8 cells with miR-146a-5p or miR-146b-5p mimics or inhibitors. Compared with the control group, overexpression of miR-146a-5p or miR-146b-5p inhibited the luciferase activity of wild-type TRAF6 3'-UTR. Furthermore, this inhibition was rescued by both miR-146a-5p and miR-146b-5p binding site mutations (Figure 5D-E). Conversely, co-transfection of miR-146a-5p or miR-146b-5p inhibitors significantly increased the luciferase activity of the reporter with wild-type TRAF6 3'-UTR, but not that of the mutant reporter (Figure 5D-E). In order to evaluate the effects of miR-146a-5p and miR-146b-5p on TRAF6 expression, we transfected miR-146a-5p or miR-146b-5p mimics and their inhibitors in HTR-8 and JEG3 cells. The results showed that miR-146a-5p and miR-146b-5p mimics reduced TRAF6 expression, while their inhibitors displayed opposite effects, in HTR-8 (Figure 5F-H) and JEG3 (Figure 5I-K) cells. Collectively, our results indicate that TRAF6 is the common target of miR-146a-5p and miR-146b-5p.

### MiR-146a-5p and miR-146b-5p suppress EMT, migration, and invasion of trophoblasts by downregulating TRAF6 expression

**Figure 6:**
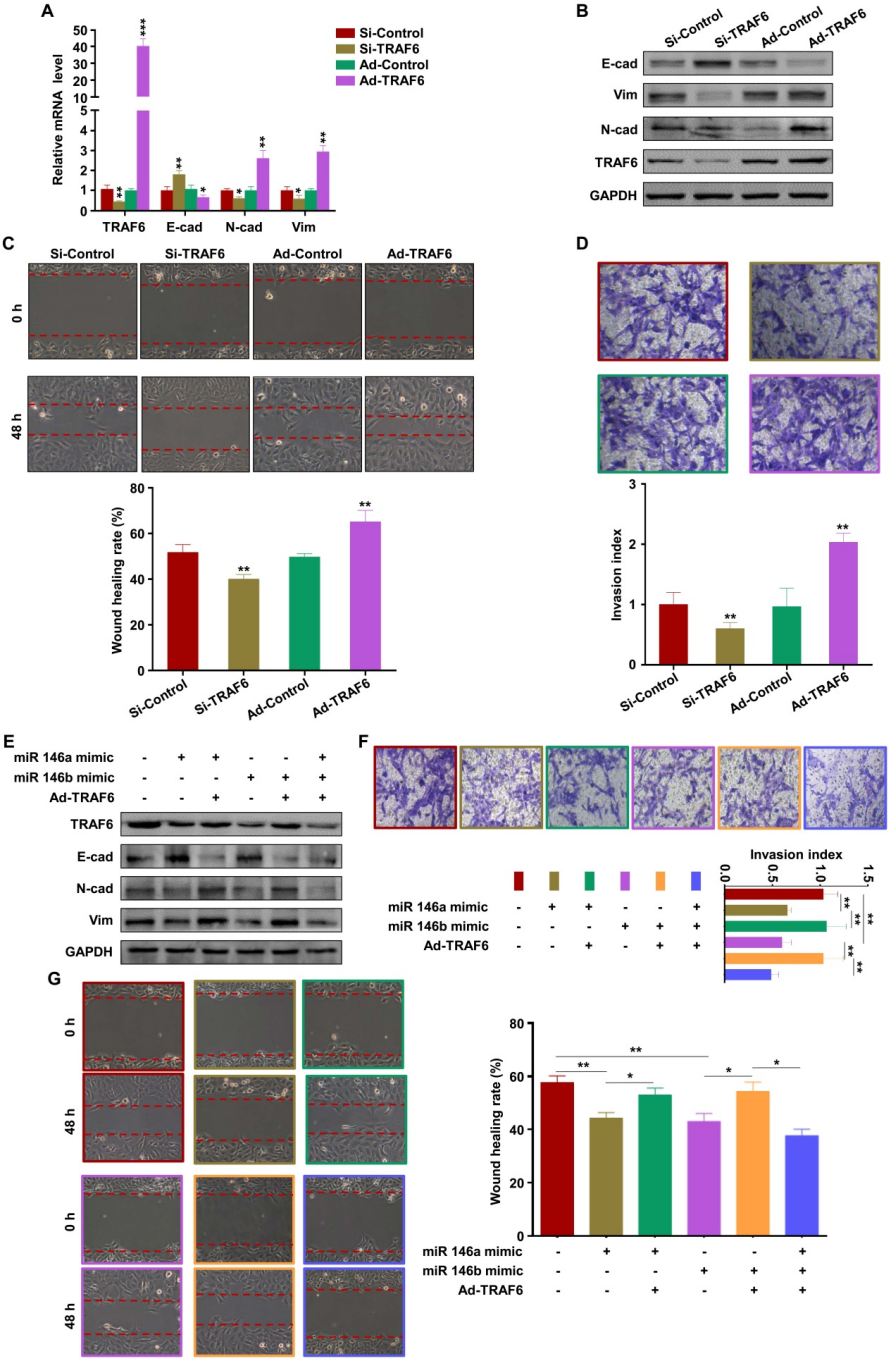
** miR-146a-5p and miR-146b-5p suppress EMT, migration, and invasion of HTR-8 by downregulating TRAF6 expression.** (**A,B**) Levels of TRAF6, E-cadherin (E-cad), N-cadherin (N-cad), and vimentin (Vim) mRNA and protein measured by RT-PCR and western blotting in HTR-8 cells 48 h after transfection with si-TRAF6 or Ad-TRAF6. (**C,D**) Migration and invasion capacities of HTR-8 cells transfected with si-TRAF6 or Ad-TRAF6 determined by wound healing and transwell assays, respectively. Representative images of migrated or invaded cells are shown (magnification, ×200). (**E**) Western blotting analysis of HTR-8 cells 72 h after transfection with miR-146a-5p mimic alone or in combination with miR-146b-5p mimic or Ad-TRAF6. (**F,G**) Migration and invasion capacities of HTR-8 cells determined by wound healing and transwell assays, respectively. Representative images of migrated or invaded cells are shown (magnification, × 200). Error bars, SD. *P < 0.05, **P < 0.01, *** P < 0.001.

The effect of TRAF6 on trophoblasts was analyzed by transfection with si-TRAF6 and Ad-TRAF6. Western blotting and RT-PCR showed that TRAF6 expression was downregulated by si-TRAF6 and upregulated by Ad-TRAF6 in HTR-8 cells (Figure 6A-B). Ad-TRAF6 upregulated N-cadherin and vimentin and downregulated E-cadherin at the mRNA and protein levels (Figure 6A-B). The invasion and migration abilities of HTR-8 cells decreased significantly after TRAF6 knockdown, while TRAF6 overexpression promoted migration and invasion (Figure 6C-D). Similar results were obtained in JEG3 cells (Figure S6A-D). In addition, Ad-TRAF6 rescued the inhibitory effect of miR-146a-5p mimic on the expression of TRAF6, N-cadherin, and vimentin and inhibited the upregulation of E-cadherin by miR-146a-5p mimic in HTR-8 (Figure 6E) and JEG3 (Figure S6E) cells. Similar results were observed for miR-146b-5p mimic (Figure 6E, Figure S6E). Interestingly, co-transfection of miR-146a-5p and miR-146b-5p mimics revealed a stronger effect than either single transfection (Figure 6E, Figure S6E). Ad-TRAF6 co-transfection rescued the inhibited invasion and migration of HTR-8 cells by miR-146a-5p or miR-146b-5p mimics (Figure 6F-G). Similar results were obtained in JEG3 (Figure S6F-G) cells. Altogether, these results indicate that miR-146a-5p and miR-146b-5p suppress EMT, migration, and invasion of trophoblasts by downregulating TRAF6 expression.

### M1-EVs promote embryos abortion by transferring miR-146a-5p and miR-146b-5p

**Figure 7:**
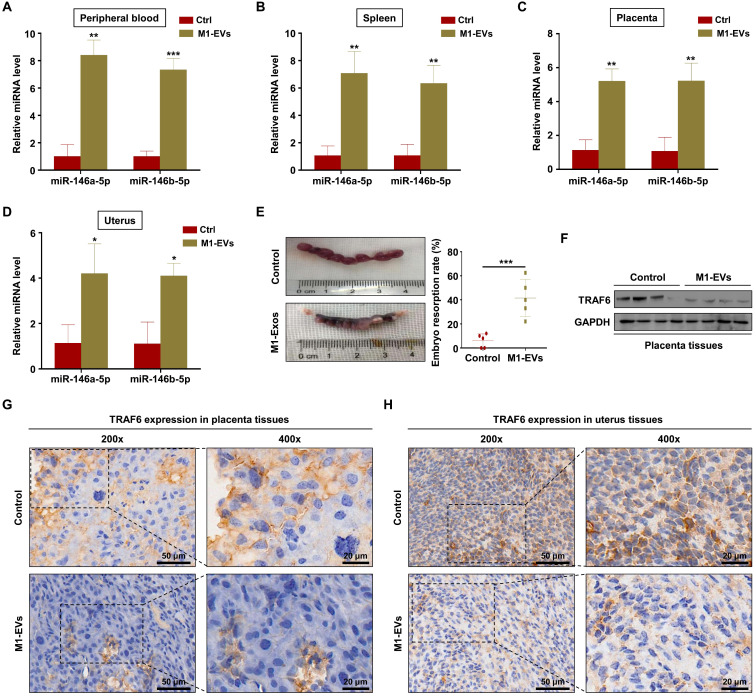
** M1-EVs promote embryo abortion by transferring miR-146a-5p and miR-146b-5p*.***M1-EVs were injected into female C57BL/6 mice via the tail vein on days 0.5 (the day of vaginal plug detection), 3.5, and 7.5. (**A-D**) Expression levels of miR-146a-5p and miR-146b-5p in the peripheral blood, spleen, placenta, and uterus measured by RT-PCR on day 11.5. (**E**) Embryo absorption rate in the M1-EVs and control groups. (**F**) Expression level of TRAF6 protein in the placenta measured by western blotting. (**G,H**) Distribution of TRAF6 in the placenta and uterus detected using IHC. Scale bars: 200 ×, 50 nm; 400 ×, 20 nm. Error bars, SD.

To verify the role of M1-EVs *in vivo*, EVs were extracted from the supernatants of Raw264.7 macrophages stimulated with LPS and IFN-γ. Female C57BL/6 mice mated with male BALB/c mice were injected with M1-EVs or PBS via the tail vein. The expression levels of miR-146a-5p and miR-146b-5p in the peripheral blood and spleen were measured by RT-PCR. The results confirmed that miR-146a-5p and miR-146b-5p were overexpressed in mice injected with M1-EVs (Figure 7A-B). miR-146a-5p and miR-146b-5p were similarly overexpressed in the uterus and placenta (Figure 7C-D). Administration of M1-EVs also significantly increased the embryo resorption rate (Figure 7E). Furthermore, the expression level of TRAF6 was markedly reduced in the placentas of mice injected with M1-EVs (Figure 7F). IHC was used to assess the distribution of TRAF6 in placental and uterine tissues. The staining showed that TRAF6 was localized in the cell membrane and plasma of both placenta and uterus tissues, which mainly observed in longitudinal muscle layer, circular muscle layer and stroma of spongiotrophoblast. Importantly, the expression level of TRAF6 was markedly reduced in both placenta and uterus tissues from mice injected with M1-EVs (Figure 7G-H). Taken together, these data suggest that M1-EVs aggravate embryo abortion by transferring miR-146a-5p and miR-146b-5p to suppress expression of TRAF6.

### MiR-146a-5p and miR-146b-5p are upregulated in placental villous tissues from patients with RSA

**Figure 8:**
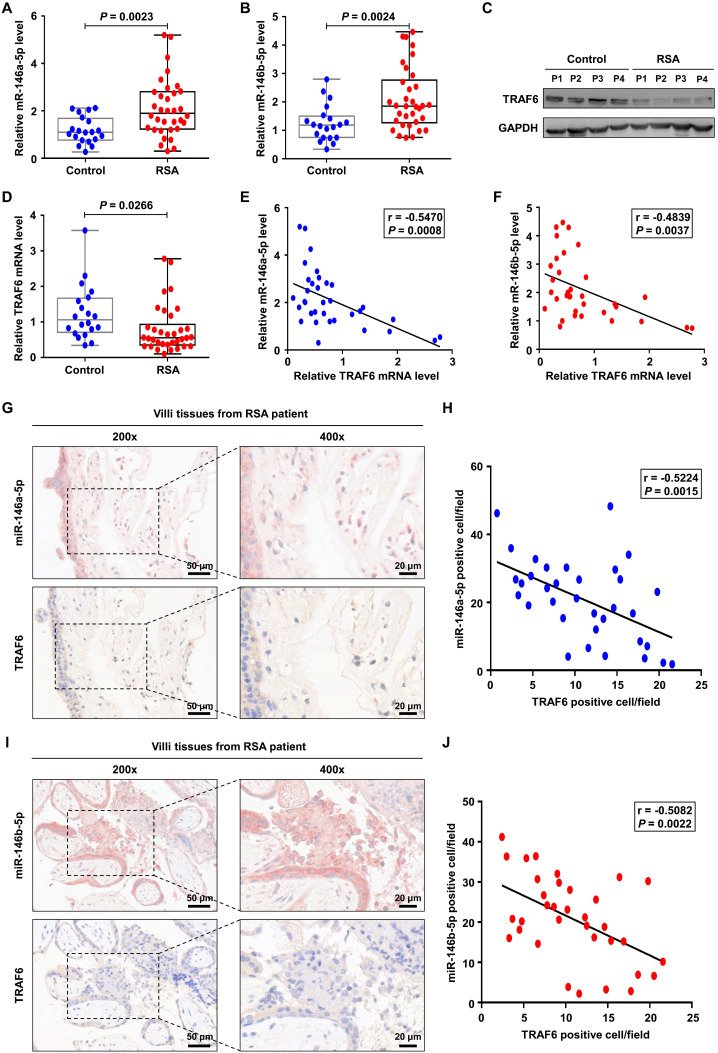
** miR-146a-5p and miR-146b-5p are upregulated in placental villous tissues from patients with RSA.** (**A,B**) Expression levels of miR-146a-5p and miR-146b-5p measured by RT-PCR in the placental villous tissues of patients with RSA (n = 34) and control patients (n = 20). (**C,D**) Expression levels of TRAF6 protein and mRNA in the placental villous tissues measured by western blotting and RT-PCR, respectively. (**E**) Negative correlation between TRAF6 and miR-146a-5p expression levels in the placental villous tissues of patients with RSA (n = 34; r = -0.547, *P* = 0.001). (**F**) Negative correlation between TRAF6 and miR-146b-5p expression levels in the placental villous tissues of patients with RSA (n = 34; r = -0.527, *P* = 0.002). (**G**) Distributions of miR-146a-5p and TRAF6 in the placental villous tissues of patients with RSA detected by ISH and IHC, respectively. (**H**) Negative correlation between TRAF6 and miR-146a-5p expression levels in the placental villous tissues of patients with RSA (n = 34; r = -0.522, *P* = 0.003). (**I**) Distributions of miR-146b-5p and TRAF6 in the placental villous tissues of patients with RSA detected by ISH and IHC, respectively. (**J**) Negative correlation between TRAF6 and miR-146b-5p expression levels in the placental villous tissues of patients with RSA (n = 34; r = -0.508, *P* = 0.002). Scale bars: 200 ×, 50 nm; 400 ×, 20 nm. Error bars, SD.

Finally, we investigated the expression levels of miR-146a-5p, miR-146b-5p, and TRAF6 in placental villous tissues from 34 patients with RSA and twenty control patients. Increased expression levels of miR-146a-5p and miR-146b-5p were observed in the samples from patients with RSA (Figure 8A-B), accompanied by decreased expression of TRAF6 at the protein and mRNA levels (Figure 8C-D). Negative correlations between the expression levels of miR-146a-5p and TRAF6 (r = -0.547, *P* = 0.001) as well as miR-146b-5p and TRAF6 (r = -0.527, *P* = 0.002) were identified in patients with RSA (Figure 8E-F). In addition, ISH and IHC were used to assess the distributions of miR-146a-5p, miR-146b-5p, and TRAF6 in patient tissues. miR-146a-5p and miR-146b-5p were mainly located on the cell membrane, while TRAF6 was also located in the plasma (Figure 8G, I). Negative correlations between the expression levels of miR-146a-5p and TRAF6 (r = -0.522, *P* = 0.003) as well as miR-146b-5p and TRAF6 (r = -0.508, *P* = 0.002) were again identified in patients with RSA (Figure 8H, J). Together, these clinical data suggest that miR-146a-5p and miR-146b-5p are significantly associated with the occurrence and development of RSA.

## Discussion

In this study, we demonstrated that EVs derived from M1-Mφ inhibit EMT, migration, and invasion of trophoblasts by transferring miR-146a-5p and miR-146b-5p targeting the expression of TRAF6. This suggests that miR-146a-5p and miR-146b-5p-enriched EVs derived from M1-Mφ might contribute to the pathogenesis of RSA. To the best of our knowledge, this is the first study to analyze the effect of macrophage-derived EV miRNA on trophoblasts and its role in RSA.

The roles of insufficient trophoblast migration and invasion in the pathogenesis of RSA have been explored by numerous studies 2, 5, 9. As part of the trophoblast differentiation process, EMT occurs during trophoblast invasion 11, 12, and defective EMT in placental trophoblasts is one of the pathologies associated with pregnancy complications 10, 13, 42, 43. Recent studies have revealed some molecular mechanisms that regulate trophoblast EMT, migration, and invasion at the maternal-fetal interface 17, 44. As the second largest decidual leukocyte population in early pregnancy, macrophages play important roles in regulation of trophoblast biological functions 3, 45-47. Macrophages can be divided into M1 and M2 subtypes, and ours and other studies have confirmed the abnormal distribution of M1-Mφ in RSA 3, 4, 20, 48, 49. A previous study indicated that activated macrophages affect trophoblast function and placental development, which may result in various adverse pregnancy outcomes 50. Several possible molecules are involved in the effect of M1-Mφ on trophoblast migration and invasion, such as TNF-α, IL-10, TGF-β, and NO 14, 15, 46, 47. In recent years, numerous studies have shown that M1-Mφ reduce tumor progression and inhibit tumor growth by promoting the Th1 response and secretion of ROS 51, 52, and repolarization of tumor-associated macrophages toward M1 phenotype is an active area of research for antitumor therapy 16. Therefore, repolarization of M1-Mφ or interference of molecules secreted by M1-Mφ may be potential treatments for pregnancy complications associated with insufficient trophoblast invasion.

EVs act as messengers between fetus and mother. In one direction, active substances from embryo-derived EVs are transferred to cells of the maternal immune and vascular systems 53, and inflammation signals delivered by embryonic EVs may contribute to the onset of parturition and labor 53, 54. In the other direction, EVs released from the mother support the growth and survival of the fetus. The concentration of EVs in maternal peripheral blood is much more abundant than that observed in non-pregnant women 55. In healthy pregnant women, the concentration of placenta-derived EVs in maternal plasma increases as gestation progresses, which reaches a peak at term 56. Studies on EV biomarkers have been carried out in pregnancy complications such as preeclampsia and hypertension 57, 58, and EV miR-100-5p, miR-378d, and miR-215-5p are promising biomarkers for early ectopic pregnancy 59. This study demonstrated that EVs derived from M1-Mφ might participate in the pathological process of RSA by affecting EMT, migration, and invasion of trophoblasts.

miRNAs, the post-transcriptional regulators of gene expression, were reported to regulate 60% of human genes by inhibiting translation of the target mRNAs or inducing mRNA degradation 60. As miRNAs are one of the most abundant biologically active substances in EVs, analysis of EV miRNA could give information about the status of the producer cell and the influenced gene expression pattern of the recipient cells 61. Mounting evidence has shown that miRNAs transferred by EVs regulate homeostasis and disorders in pregnancy 27. For instance, EV miR-517-3p secreted by trophoblasts inhibited the activation and proliferation of T and NK cells 62, and EV miR-141 suppressed T cell proliferation and contributed to the mechanisms of maternal tolerance 63. The roles of miRNAs in the pathological process of RSA have been confirmed by numerous studies in recent years. Zhang et al. confirmed the abnormal expression of miR-184 in RSA, and miR-184 promoted apoptosis of trophoblasts via targeting WIG1 64. A study by Gu et al. suggested that miR-3074-5p contributes to the pathogenesis of RSA by promoting apoptosis of trophoblasts and inhibiting their invasion 65. Our previous study showed that miR-27a-3p might contribute to EMT of trophoblasts via targeting USP25, thereby inhibiting their migration and invasion 2. A study from Liu et al. suggested that miR-93 regulates trophoblast proliferation, migration, invasion, and apoptosis by targeting expression of BCL2L2 and is involved in the pathogenesis of RSA 66. Previous studies have demonstrated that miR-146a-5p regulates the proliferation and apoptosis of trophoblasts 67 and has an anti-inflammatory effect on trophoblasts 68. Although studies have identified abnormal expression of miR-146a 69 and miR-146b-5p 70 in RSA, the underlying mechanisms have not been expounded. Our study provides evidence that M1-EVs shuttle miR-146a-5p and miR-146b-5p into trophoblasts and that miR-146a-5p and miR-146b-5p overexpression inhibit the invasion and migration of trophoblasts *in vitro*, thereby exerting a negative regulatory role in trophoblast EMT.

TRAF6, a member of the TRAF family, is a type of adaptor protein and E3 ubiquitin ligase 71. TRAF6 is regarded as an amplified oncogene in human lung cancer 72 and promotes angiogenesis by upregulating HIF-1α 73. Knockdown of TRAF6 was found to decrease the invasion and metastasis abilities of melanoma and lung cancer 74, 75. It has been reported that TRAF6 plays important roles in pregnancy-induced epithelial cell expansion 76. Zhao et al. showed that miR-643 inhibits endometritis progression by downregulating TRAF6, suggesting a possible role of TRAF6 in endometritis 77. Our results suggest that TRAF6 promotes EMT, migration, and invasion of trophoblasts, and miR-146a-5p and miR-146b-5p derived from EVs of M1-Mφ participate in trophoblast regulation by targeting TRAF6.

## Conclusions

**Figure 9:**
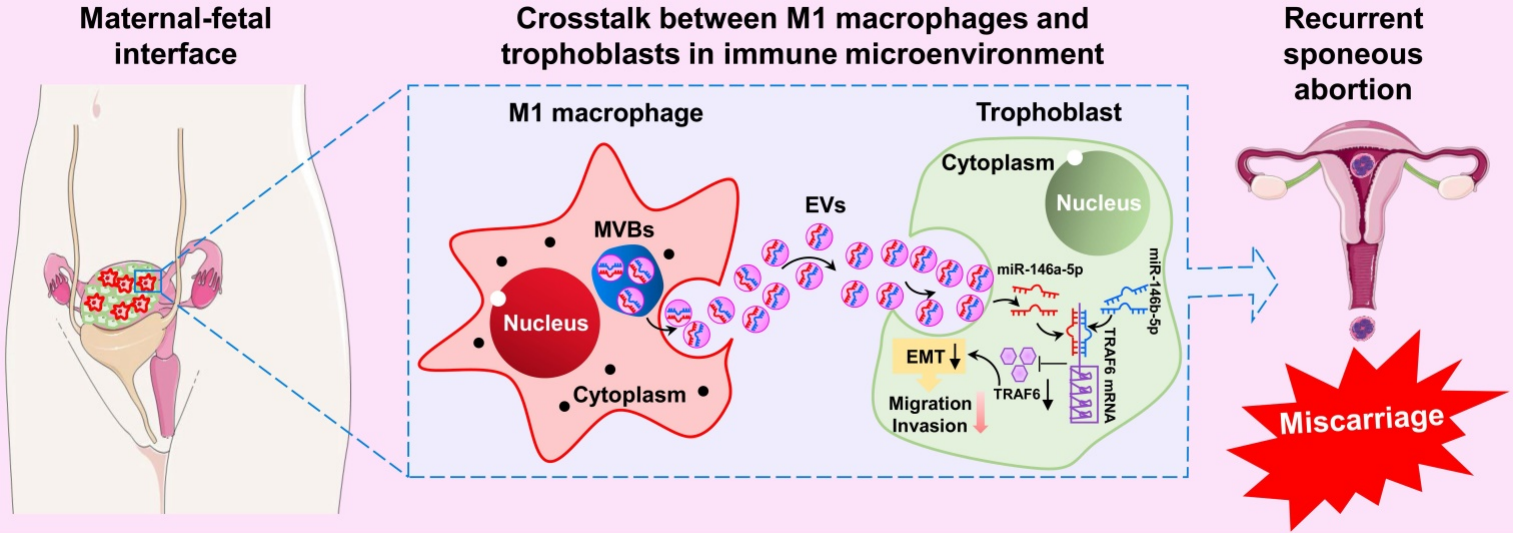
** Schematic illustration of M1-Mφ-derived miR-146a-5p and miR-146b-5p inhibition of trophoblast EMT, migration, and invasion in RSA.** Extracellular vesicles derived from M1-Mφ suppress EMT, migration, and invasion of trophoblasts by transporting miR-146a-5p and miR-146b-5p to directly inhibit TRAF6 expression at the post-transcriptional level, thereby participating in the pathogenesis of RSA.

In summary, our results show that M1-Mφ-derived EVs suppress EMT, migration, and invasion of trophoblasts by transporting miR-146a-5p and miR-146b-5p to directly inhibit TRAF6 expression at the post-transcriptional level, thereby participating in the pathogenesis of RSA (Figure 9). These findings illustrate a new dialogue between macrophages and trophoblasts in the microenvironment of the maternal-fetal interface, which represents a novel mechanism of M1-Mφ regulation of EVTs in RSA. These findings highlight EV miR-146a-5p and miR-146b-5p as promising therapeutic targets for RSA. However, further studies are needed to extend the current results in cell lines.

## Supplementary Material

Supplementary figures and tables.

## Abbreviations

Ad-TRAF6: adenovirus for TRAF6
EMT: epithelial-mesenchymal transition
EVs: extracellular vesicles
EVTs: extravillous trophoblasts
IHC: immunohistochemistry
ISH: *in situ* hybridization
M1-EVs: EVs from M1 macrophages
M1-Mφ: M1 macrophages
M2-Mφ: M2 macrophages
NTA: nanoparticle tracking analysis
PBS: phosphate-buffered saline
RSA: recurrent spontaneous abortion
RT-PCR: quantitative real-time PCR
TEM: transmission electron microscopy
TRAF6: TNF receptor-associated factor 6
