# Supplementary material for: Extracellular vesicles derived from M1 macrophages deliver miR-146a-5p and miR-146b-5p to suppress trophoblast migration and invasion by targeting TRAF6 in recurrent spontaneous abortion
Source: Theranostics. 2021 Mar 31;11(12):5813–30. doi: 10.7150/thno.58731 (PMC8058722; doi:10.7150/thno.58731)
Supplement: Supplementary file 1 — Supplementary figures and tables. [file thnov11p5813s1.pdf]

Supplementary Table

Table S1. Comparison of the Baseline Data between Control Group and RSA Group.

Parameter	Control (n = 20)	RSA (n = 34)
Age (years)	30.16±2.83	30.28±4.23
BMI (kg/m^2)	21.57±2.66	21.40±4.61
Gestation week	8.53±1.38	9.02±1.18
Number of miscarriage	0.72±0.20	2.45±0.32**
Number of live birth	1.62±0.40	0.00±0.00**

Notes: ***P*<0.01. Abbreviations: RSA, recurrent spontaneous abortion; BMI, body mass index.

Table S2. The Sequences of Primers for Quantitative RT-PCR.

Gene	Primer Sequence
IL-6	F：5`-ACTCACCTCTTCAGAACGAATTG -3`<br>R：5`-CCATCTTTGGAAGGTTCAGGTTG-3`
TNF-α	F：5`-TCTCGAACCCCGAGTGACAA-3`<br>R：5`-TGAAGAGGACCTGGGAGTAG-3`
IFN-β	F：5`-ATGACCAACAAGTGTCTCCTCC-3`<br>R：5`-GGAATCCAAGCAAGTTGTAGCTC-3`
N-cadherin	F：5`-TCAGGCGTCTGTAGAGGCTT-3`<br>R：5`-ATGCACATCCTTCGATAAGACTG -3`
E-cadherin	F：5`-ATTTTTCCCTCGACACCCGAT-3`<br>R：5`- TCCCAGGCGTAGACCAAGA-3`
vimentin	F：5`-AGTCCACTGAGTACCGGAGAC-3`<br>R：5`- CATTTCACGCATCTGGCGTTC-3`
GAPDH	F：5`-GCACCACCAACTGCTTAGCA-3`<br>R：5`-GTCTTCTGGGTGGCAGTGATG-3`

Table S3. The expression abundance of miRNAs in M1-EVs.

miRNA profile	Sample 1	Sample 2	Sample 3
hsa-miR-146a-5p	938617	431209	471598
hsa-miR-92a-3p	638750	106484	50822
hsa-miR-24-3p	285214	76071	51707
hsa-miR-146b-5p	133509	68596	56170
hsa-miR-21-5p	274093	48741	28220
hsa-miR-221-3p	176843	42658	37752
hsa-miR-378a-3p	97312	29625	50595
hsa-miR-222-3p	163596	20344	9609
hsa-miR-148a-3p	78001	17957	28991
hsa-miR-320a-3p	87061	21200	10762
hsa-miR-27a-3p	80344	16448	12655
hsa-miR-23a-3p	40452	12561	23690
hsa-miR-155-5p	41894	17151	16865
hsa-miR-30d-5p	46256	18951	8290
hsa-miR-423-5p	81411	12756	3611
hsa-miR-1246	10548	4779	26731
hsa-let-7a-5p	68696	7603	5012
hsa-miR-25-3p	41061	9156	7320
hsa-miR-30c-5p	23617	8874	10089
hsa-let-7i-5p	33691	7468	6867

Supplementary Figure

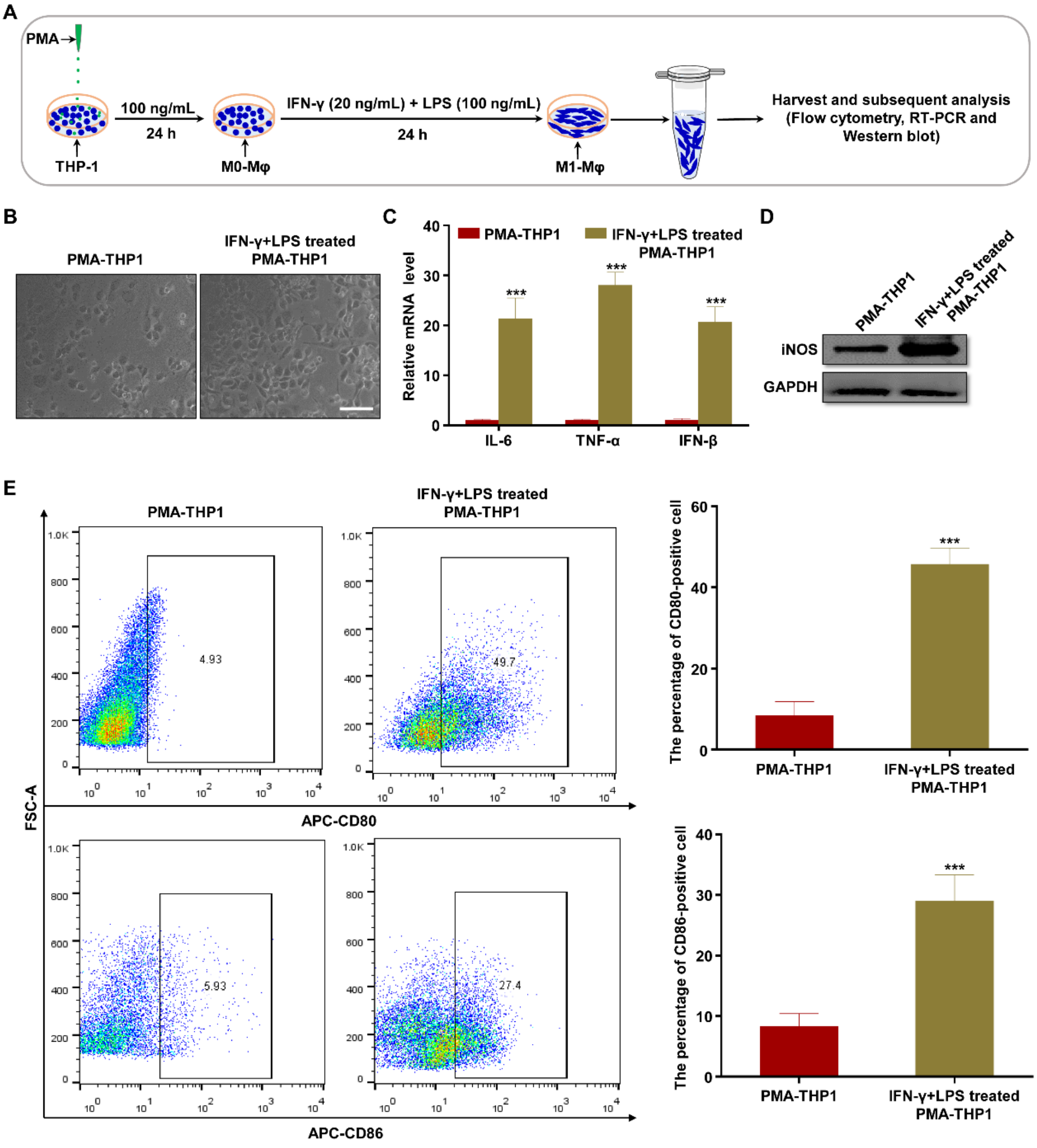

Figure S1. Induction and identification of M1 macrophages. (A) Schematic diagram of M1 macrophage induction process. THP-1 cells were cultured in 50 ng/mL PMA for 24 h, and then stimulated with 100 ng/mL LPS plus 20 ng/mL IFN-γ for 24 h. (B) The representative bright-field images of macrophages treated by the respective conditioned media are shown (magnification, × 200). (C) RT-PCR assays of IL-6, TFN-α and IFN-β mRNAs in PMA-THP1 and IFN-γ + LPS-treated PMA-THP1. (D) Western blotting analysis of iNOS protein levels in PMA-THP1 and IFN-γ + LPS-treated PMA-THP1. (E) Flow cytometry for analyzing the expression of CD80 and CD86 in PMA-THP1 and IFN-γ + LPS-treated PMA-THP1. Error bars, SD. ***P < 0.001.

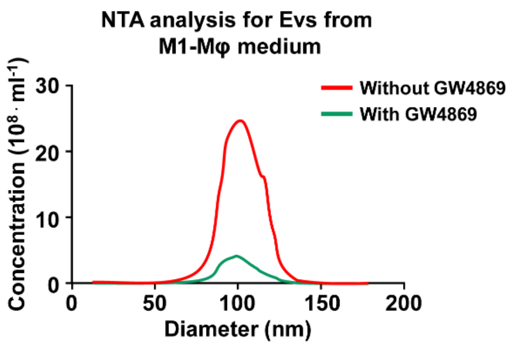

Figure S2. NTA of the size distribution and concentration of EVs from equal volume of culture medium of M1-Mφ pretreated with or without GW4869.

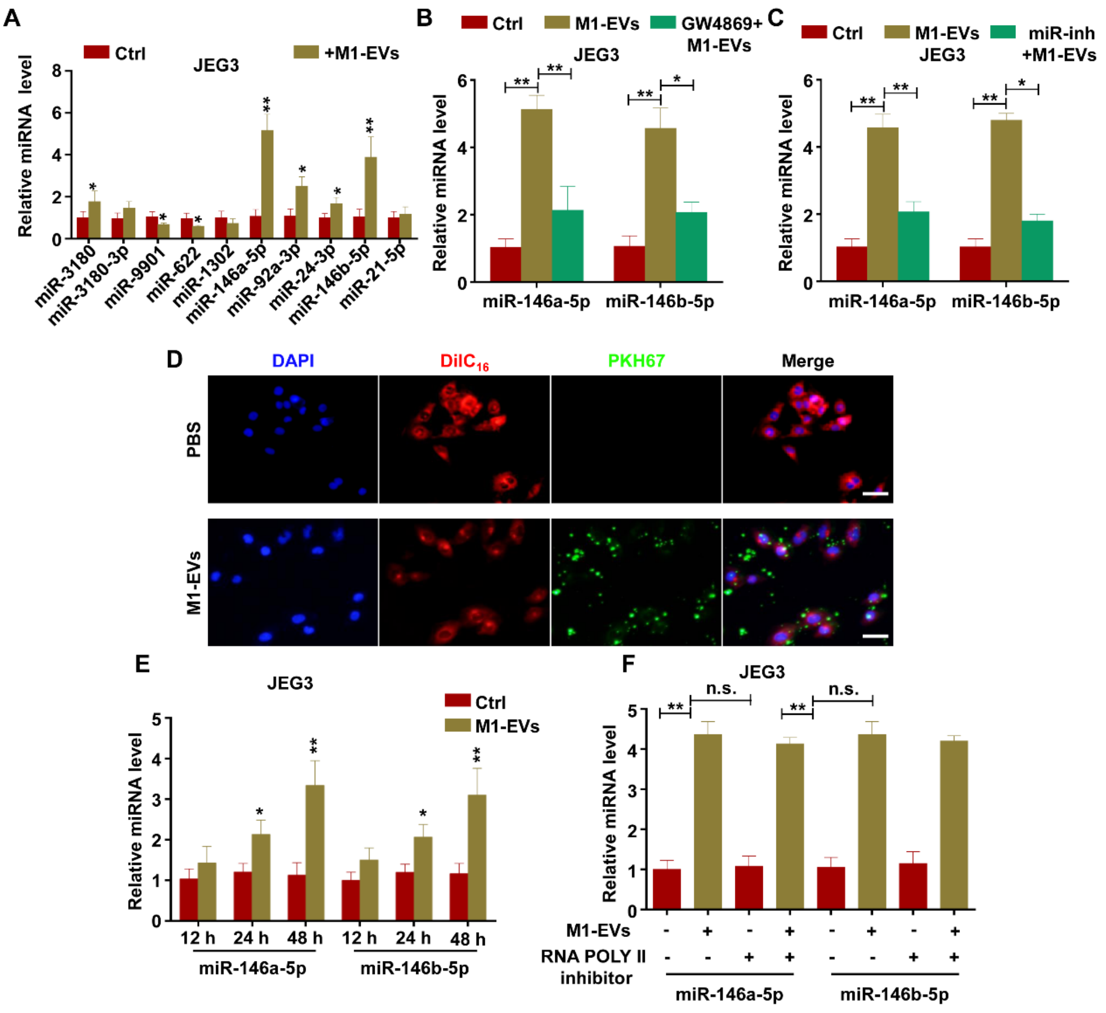

Figure S3. M1-EVs transport miR-146a-5p and miR-146b-5p into JEG3. (A) The expression of miR-146a-5p and miR-146b-5p detected by RT-PCR in JEG3, JEG3 treated with M1-EVs or EVs from M1-Mφ treated with GW4869. (B) The expression of miR-146a-5p and miR-146b-5p were detected by RT-PCR in JEG3, JEG3 treated with M1-EVs or EVs from M1-Mφ treated with inhibitors of miR-146a-5p or miR-146b-5p. (C) JEG3 was incubated with PKH67-labeled EVs from M1-Mφ for 12 h, 24 h and 48 h. The green EVs signal was detected by confocal microscopy (scale bar, 20 μm). (D) The expression of miR-146a-5p and miR-146b-5p were detected by RT-PCR. (E) JEG3 were treated with polymerase II inhibitors (20 mM) for 3 h and then incubated with M1-EVs. Levels of miR-146a-5p and miR-146b-5p were assessed by RT-PCR. Error bars, SD. *$P < 0.05$, **$P < 0.01$; n.s., not significant.

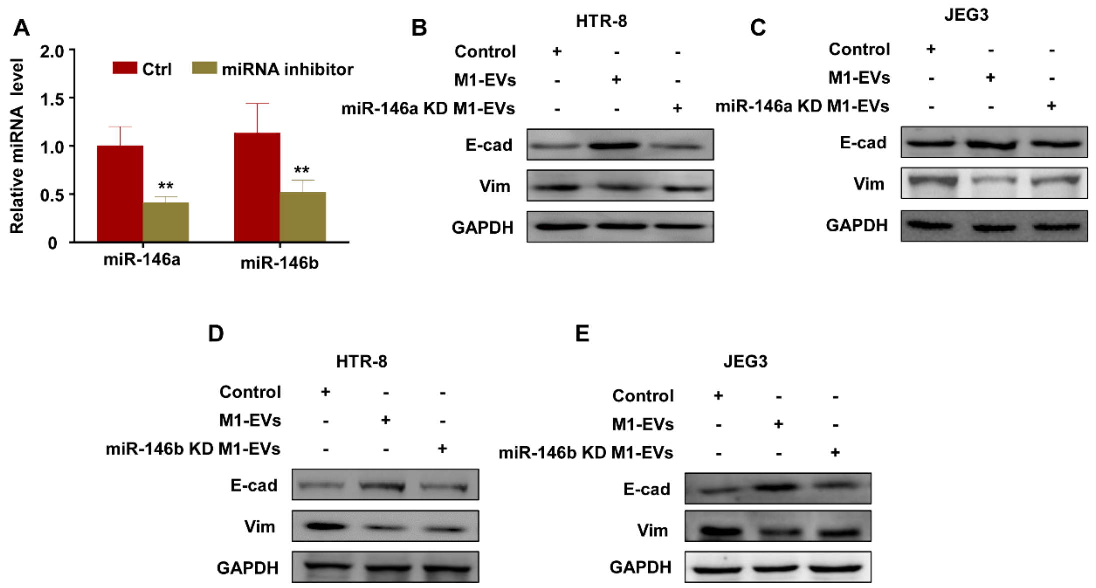

Figure S4. The effect of miR-146a-5p and miR-146b-5p knockdown in M1-Mφ on EMT of trophoblasts. (A) The expression of miR-146a-5p and miR-146b-5p detected by RT-PCR in M1-Mφ treated with corresponding adenovirus. (B-C) Levels of E-cad and Vim protein were analyzed by western blotting at 48 h post-transfection. (D-E) Levels of E-cad and Vim protein were analyzed by western blotting at 48 h post-transfection. Error bars, SD. $^{**}P < 0.01$.

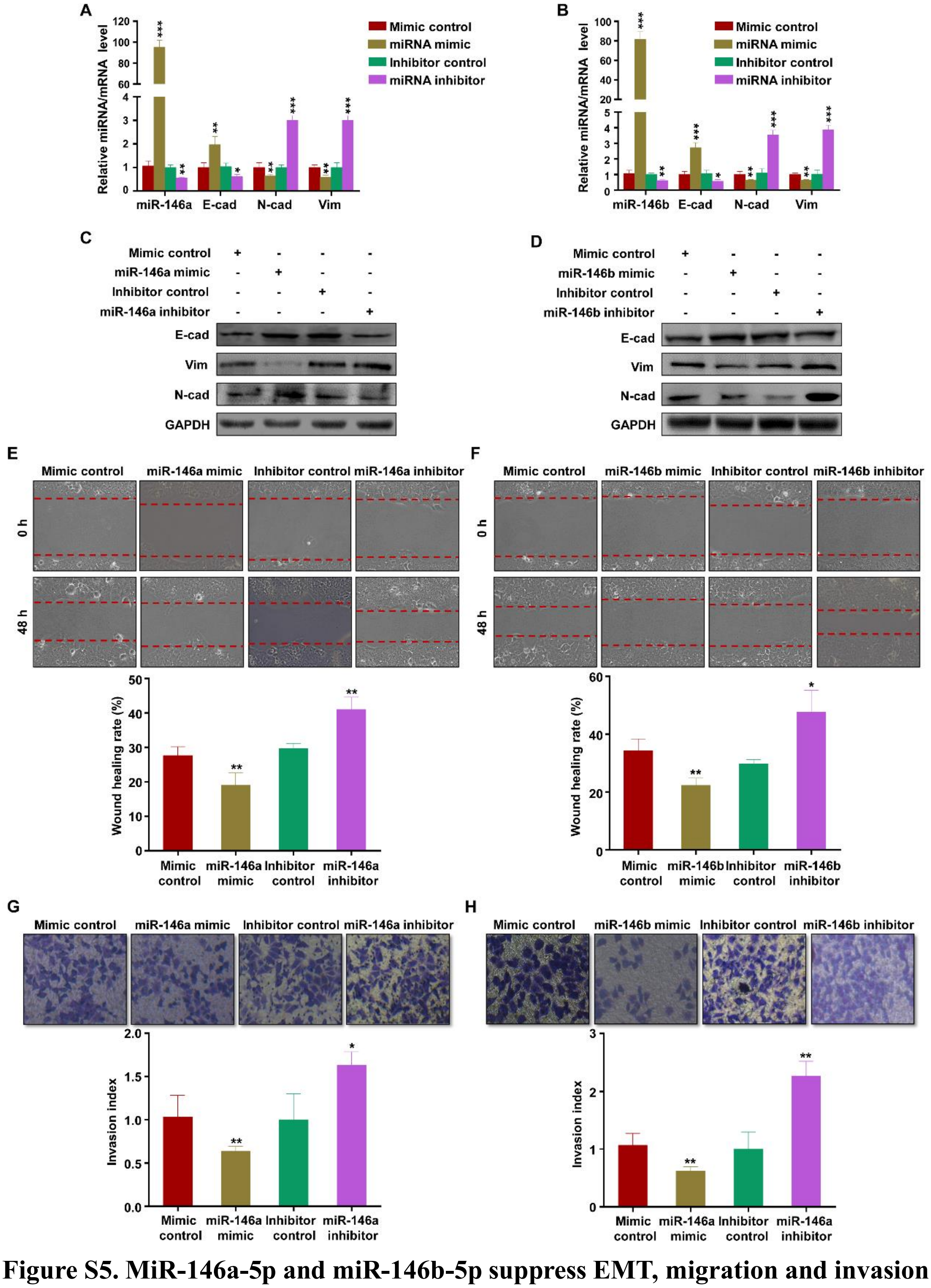

Figure S5. MiR-146a-5p and miR-146b-5p suppress EMT, migration and invasion of JEG3. (A-B) JEG3 was transfected with miR-146a-5p or miR-146b-5p mimics or inhibitor, respectively. The levels of miR-146a-5p or miR-146b-5p, and E-cadherin (E-cad), N-cadherin (N-cad), and vimentin (Vim) were analyzed by RT-PCR at 48 h post transfection. (C-D) Levels of E-cad, N-cad and Vim protein were analyzed by western blotting at 48 h post-transfection. (E-H) The migration and invasion capacity of JEG3 alone or co-cultured with M1-EVs was determined by the wound healing assay and

transwell coculture system, respectively. Representative photographs of migratory or invaded cells (magnification, × 200) are shown. Error bars, SD. *$P < 0.05$, **$P < 0.01$, ***$P < 0.001$.

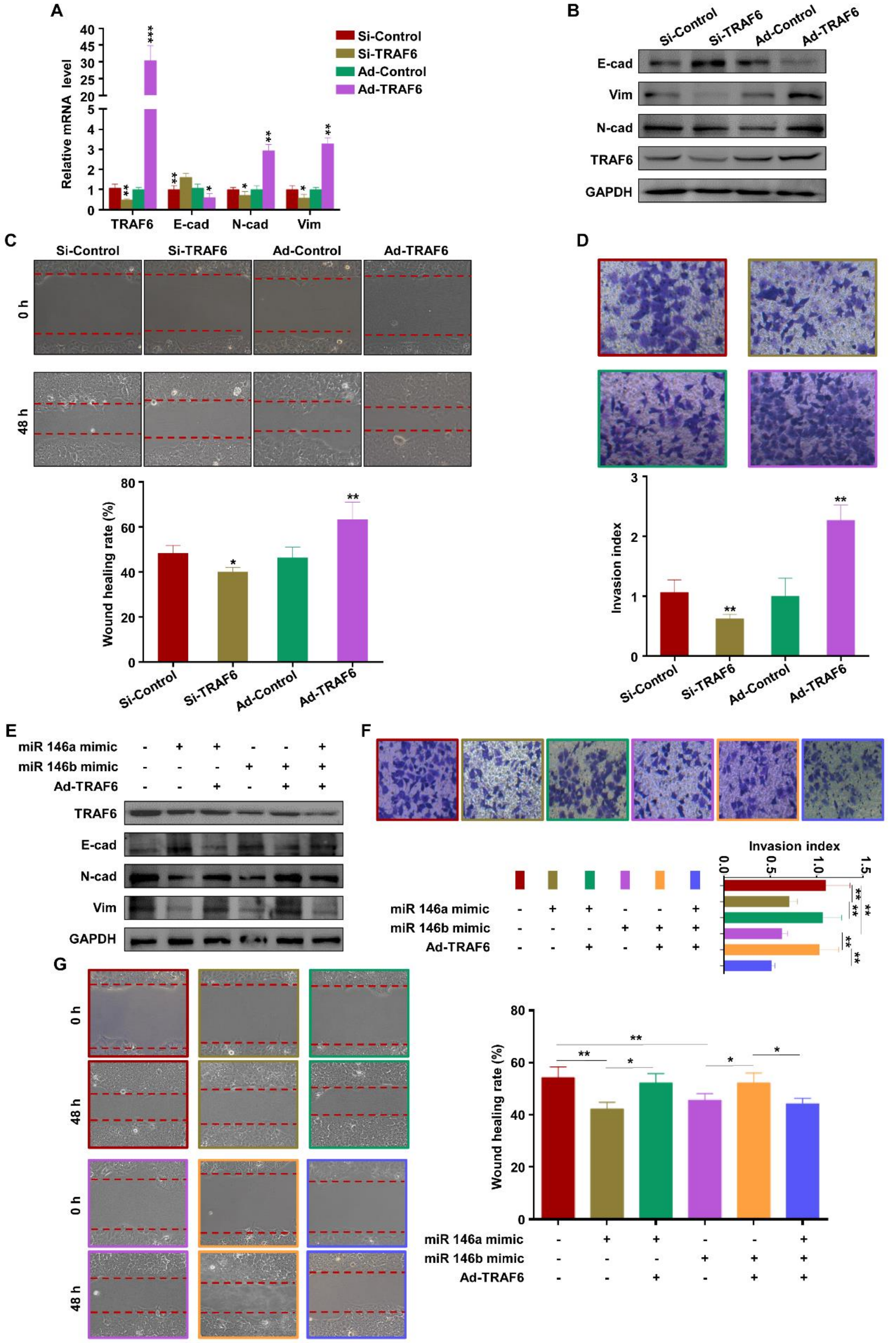

Figure S6. MiR-146a-5p and miR-146b-5p suppress EMT, migration and invasion of JEG3 by down-regulating TRAF6 expression. (A) JEG3 was transfected with si-TRAF6 or Ad-TRAF6, respectively. The levels of TRAF6, E-cadherin (E-cad), N-cadherin (N-cad), and vimentin (Vim) were analyzed by RT-PCR at 48 h post-transfection. (B) Levels of TRAF6 protein in JEG3 were analyzed by western blotting at 48 h post-transfection. (C-D) The migration and invasion capacity of JEG3 transfected with si-TRAF6 or Ad-TRAF6 was determined by the wound healing assay and transwell co-culture system, respectively. Representative photographs of migratory or invaded cells (magnification, × 200) are shown. (E) JEG3 transfected with miR-146a-5p mimics alone or in combination with miR-146a-5p or Ad-TRAF6 were subjected to western blotting at 72 h post-transfection. (F-G) The migration and invasion capacity of JEG3 was determined by the wound healing assay and transwell co-culture system, respectively. Representative photographs of migratory or invaded cells (magnification, × 200) are shown. Error bars, SD. *$P < 0.05$, **$P < 0.01$, ***$P <$ 0.001.
